# Survival prediction in patients undergoing radionuclide therapy based on intratumoral somatostatin-receptor heterogeneity

**DOI:** 10.18632/oncotarget.12402

**Published:** 2016-10-02

**Authors:** Rudolf A. Werner, Constantin Lapa, Harun Ilhan, Takahiro Higuchi, Andreas K. Buck, Sebastian Lehner, Peter Bartenstein, Frank Bengel, Imke Schatka, Dirk O. Muegge, László Papp, Norbert Zsótér, Tobias Große-Ophoff, Markus Essler, Ralph A. Bundschuh

**Affiliations:** ^1^ Department of Nuclear Medicine, University Hospital Wrzburg, Wrzburg, Germany; ^2^ Department of Nuclear Medicine, Ludwig-Maximilians-University Munich, Munich, Germany; ^3^ Department of Nuclear Medicine, Hannover Medical School, Hannover, Germany; ^4^ Department of Nuclear Medicine, Charité - Universitätsmedizin Berlin, Berlin, Germany; ^5^ University of Applied Sciences, Hamburg, Germany; ^6^ Department of Nuclear Medicine, Medical University of Vienna, Vienna, Austria; ^7^ Mediso Medical Imaging Systems Ltd., Budapest, Hungary; ^8^ Department of Nuclear Medicine, University Medical Center Bonn, Bonn, Germany

**Keywords:** neuroendocrine tumor, tumor heterogeneity, textural parameters, SSTR-PET/CT, radiopeptide therapy

## Abstract

The NETTER-1 trial demonstrated significantly improved progression-free survival (PFS) for peptide receptor radionuclide therapy (PRRT) in neuroendocrine tumors (NET) emphasizing the high demand for response prediction in appropriate candidates. In this multicenter study, we aimed to elucidate the prognostic value of tumor heterogeneity as assessed by somatostatin receptor (SSTR)-PET/CT. 141 patients with SSTR-expressing tumors were analyzed obtaining SSTR-PET/CT before PRRT (1-6 cycles, ^177^Lu somatostatin analog). Using the Interview Fusion Workstation (Mediso), a total of 872 metastases were manually segmented. Conventional PET parameters as well as textural features representing intratumoral heterogeneity were computed. The prognostic ability for PFS and overall survival (OS) were examined. After performing Cox regression, independent parameters were determined by ROC analysis to obtain cut-off values to be used for Kaplan-Meier analysis. Within follow-up (median, 43.1 months), 75 patients showed disease progression (median, 22.2 m) and 54 patients died (median, 27.6 m). Cox analysis identified 8 statistically independent heterogeneity parameters for time-to-progression and time-to-death. Among them, the textural feature Entropy predicted both PFS and OS. Conventional PET parameters failed in response prediction. Imaging-based heterogeneity assessment provides prognostic information in PRRT candidates and outperformed conventional PET parameters. Its implementation in clinical practice can pave the way for individualized patient management.

## INTRODUCTION

Over the past decades, a rising incidence of neuroendocrine tumors (NET) has been reported [1]. With a delay in diagnosis of 5-7 years, NET typically present at advanced stages. In clinical routine, physicians often have to rely on single tumor biopsies for treatment decisions and might therefore be prone to sampling bias which ultimately leads to either misdiagnosis or underestimation of therapeutic response [2, 3]. Therefore, non-invasive, whole-body assessment of tumor heterogeneity is highly desirable. Due to its ability to visualize functional alterations on a molecular level instead of pure morphological characteristics, positron emission tomography (PET) has proven its prognostic value in risk stratification for several types of cancer [4–6].

Recently, the NETTER-1 trial (ClinicalTrials.gov Identifier: NCT01578239) demonstrated significantly improved progression-free survival (PFS) for NET patients treated with the β-emitter labeled somatostatin analog ^177^Lu-DOTA-D-Phe-Tyr3-octreotate (DOTATATE) in advanced midgut NET [7–10]. Thus, rising numbers of peptide receptor radionuclide therapy (PRRT) can be expected in the next years.

As a prerequisite, pre-therapeutic somatostatin receptor (SSTR)-PET/computed tomography (CT) is mandatory to confirm adequate receptor density on the tumor cell surface [11, 12]. Consequently, the imaging agent ^68^Ga-DOTATATE was recently approved by the FDA which will also promote use of PRRT and simultaneously emphasizes the need for reliable response prediction prior to treatment initiation.

In this present multi-center trial, we aimed to elucidate the prognostic capability of intratumoral heterogeneity parameters assessed by baseline SSTR-PET/CT in patients scheduled for radionuclide therapy.

## RESULTS

A total of 141 SSTR-PET scans were performed prior to PRRT. Baseline PET was positive in all patients as a prerequisite for treatment initiation. 120/141 (85.1%) subjects suffered from liver metastases, more than half of the cohort demonstrated lymph node metastases (78/141, 55.3%), one third suffered from bone lesions (53/141, 37.6%) and 16/141 (11.3%) demonstrated pulmonary metastases (Table 1).

**Table 1:** Detailed patients’ characteristics

Characteristic		Number of subjects (%)
Sex	female male	71/141 (50.4) 70/141 (49.6)
Age (years)* Ki67 (%)* Chromogranin A (µg/l)*	64, 24-83 5, 1-40 571, 35 – 64700	
Primary	GEP-NET total	108/141 (76.6)
	pancreatic	45/141 (31.9)
	ileum/jejunum/mesenterial	51/141 (36.1)
	colon	7/141 (5.0)
	stomach	5/141 (3.5)
	cancer of unknown primary	15/141 (10.6)
	Lung	9/141 (6.4)
	other^#^	9/141 (6.4)
Previous treatment	surgical of the primary	76/141 (53.9)
	Sandostatin	79/141 (56.0)
	chemotherapy	39/141 (27.7)
	radiation therapy	15/141 (10.6)
Baseline SSTR-PET/CT	Metastases according to PET	
	liver	120/141 (85.1)
	lymph nodes	78/141 (55.3)
	bone	53/141 (37.6)
	lung	16/141 (11.3)
	Administered dose (MBq)* 124, 61-239	
PRRT information	Activity per cycle (GBq)* 7.3, 0.9-9.0	
	Number of cycles* 4, 1-6	

*= median and range is given, # = including meningeoma, hemangioendothelioma, pheochromocytoma, medullary thyroid carcinoma and pituitary tumor. GBq = Gigabecquerel, GEP-NET = gastroenteropancreatic neuroendocrine tumors, MBq = Megabecquerel, PRRT = Peptide Receptor Radionuclide Therapy, SSTR-PET/CT = somatostatin receptor positron emission tomography/computed tomography.

Within follow-up (median, 43.1 months, range, 22.3 - 89.8 m), 75/139 subjects (54.0%) experienced progressive disease. On average, progression was detected at a median of 22.2 m after the pre-therapeutic baseline PET scan (mean, 27.1 m, range, 4 days - 85.2 m). 54/141 (38.3%) patients died from their cancer after a median of 27.6 m (mean, 31.4 m, range, 4 days - 85.2 m). The median proliferation index Ki67 in those patients was 5% (range, 1-40%).

### Correlation of clinical parameters with both PFS and OS

Cox regression analysis of clinical parameters (as given in Table 1) was performed. Regarding PFS and OS, cumulative dose was the only parameter reaching significance (PFS, *p* < 0.02, OS, *p* < 0.01). Moreover, several investigated clinical features trended to be significant (Ki67, PFS, *p* = 0.09; CgA, OS, *p* = 0.06).

### Correlation of PET parameters with both PFS and OS

In Cox hazard analysis, the only parameter that showed significant correlation for both PFS (*p* = 0.02) and OS (*p* = 0.0002) was Entropy with Hazard Ratio (HR) of 0.59 and 0.35, respectively. In addition, Skewness showed significant correlation (*p* = 0.04) with OS with an HR of 0.58.

In Receiver Operating Characteristics (ROC) analysis of the independent parameters Entropy, Correlation, Short Zone Emphasis and Homogeneity demonstrated significant prognostic ability for PFS. Significant prognostic values for OS were found for Entropy, Correlation, Homogeneity, Short Zone Emphasis and Size Variation. Of the investigated conventional PET parameters, Tissue Receptor Expression (TRE) was significant for OS (*p* = 0.003), whereas all other parameters failed in response prediction. Details can be found in Table 2 and selected ROC curves are shown in Supplementary Figure a.

**Table 2:** Receiver Operating Characteristics (ROC) analysis for Progression-Free (PFS) and Overall Survival (OS) for conventional and heterogeneity positron emission tomography (PET) parameters

Parameter		for	AUC	95% CI	Sensitivity (%)	Specificity (%)	Cut-off	*p*-value
Conventional PET Parameters	**SUVmax**	PFS OS	0.53 0.50	0.45-0.62 0.42-0.59	47 43	66 69	>23.6 >25.6	0.51 0.98
	**SUVmean**	PFS OS	0.50 0.52	0.42-0.59 0.44-0.61	28 76	61 40	≤10.3 ≤12.3	0.99 0.66
	**TRE**	PFS OS	0.57 0.64	0.48-0.65 0.56-0.72	32 44	84 82	≤1637 ≤1977	0.16 0.003*
Heterogeneity Parameters^#^	**Coefficient of Variation**	PFS	0.53	0.44-0.61	48	64	>0.3559	0.58
		OS	0.53	0.45-0.62	28	90	>0.4794	0.55
	**Skewness**	PFS	0.52	0.435-0.607	28	88	>0.8465	0.66
		OS	0.52	0.44-0.6	35	87	>0.8465	0.71
	**Entropy**	PFS	0.60	0.52-0.68	37	83	≤5.6443	0.04*
		OS	0.70	0.61-0.77	65	74	≤6.1767	0.0001*
	**Homogeneity**	PFS	0.61	0.51-0.68	39	88	>0.0175	0.020*
		OS	0.67	0.62-0.78	61	78	>0.0118	0.0004*
	**Correlation**	PFS	0.64	0.55-0.72	42	86	≤0.1581	0.004*
		OS	0.69	0.6-0.77	70	68	≤0.4184	0.0001*
	**Contrast**	PFS	0.51	0.42-0.60	32	81	≤10433	0.84
		OS	0.54	0.46-0.26	35	82	≤5186.45	0.51
	**Short Zone** **Emphasis**	PFS	0.60	0.52-0.69	56	64	>0.9998	0.031*
		OS	0.62	0.53-0.70	53	78	>0.9999	0.024*
	**Size Variation**	PFS	0.59	0.5-0.68	43	83	>0.0004	0.06
		OS	0.70	0.56.0.72	44	82	≤1976.855	<0.0001*

As obtained by Cox multiparametric analysis, only the above-mentioned heterogeneity parameters were independent from each other. Of the whole cohort (n=141), 75 patients demonstrated progressive disease, 54 died. Compared to conventional parameters, heterogeneity parameters (such as Entropy, Homogeneity, Correlation, Short Zone Emphasis, Size Variation) demonstrated higher AUC values. Additionally, these imaging-based features reached a statistically significant distinction between responders and non-responders. * = statistically significant, # = independent according to Cox analysis. CI = confidence interval, PET = positron emission tomography, SUV_mean/max_ = mean/maximum standardized uptake value, TRE = Tissue Receptor Expression.

**Figure 1:**
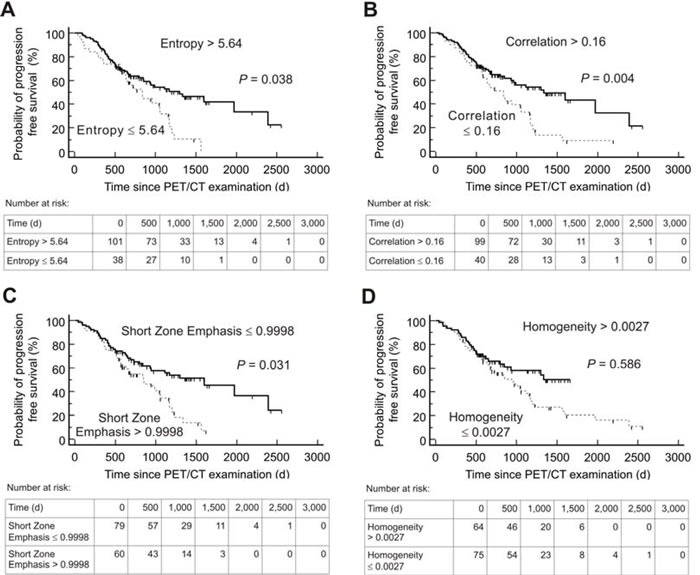
Kaplan-Meier plots and number-at-risk tables for probability of Progression-Free Survival (***n*** = 139) Low-risk group (solid lines) was identified by various textural parameters measured on somatostatin receptor positron emission tomography/computed tomography (PET/CT) before Peptide Receptor Radionuclide Therapy. Cut-off values derived by Receiver operating characteristics analysis were used. d = days.

**Figure 2:**
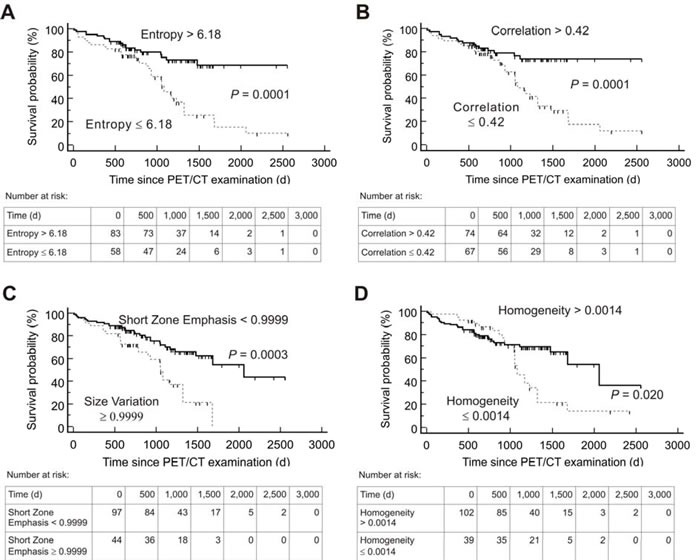
Kaplan-Meier plots and number-at-risk tables for probability of Overall Survival (***n*** = 141) Low-risk group (solid lines) was identified by various textural parameters measured on somatostatin receptor positron emission tomography/computed tomography (PET/CT) before Peptide Receptor Radionuclide Therapy. Cut-off values derived by Receiver operating characteristics analysis were used. d = days.

Kaplan-Meier analysis using the threshold evaluated by ROC revealed a significant distinction between high- and low-risk patients for both PFS and OS for the following textural parameters: Entropy, Correlation, Short Zone Emphasis and Homogeneity. In addition, TRE showed a significant distinction for OS. Details including the negative and positive hazard ratios can be found in Table 3, respective Kaplan-Meier-plots for PFS are given in Figure 1 and for OS in Figure 2. An overview of the respective AUC values for both standard and heterogeneity PET parameters regarding PFS and OS are given in Table 2.

**Table 3:** Results of Kaplan-Meier analysis regarding for Progression-Free (PFS) and Overall Survival (OS)

Parameter	for	x^2^	*p*-value	HR negative	CI	HR positive	CI
**Entropy**	PFS	7.14	0.007	1.87	1.09-3.19	0.54	0.31-0.92
	OS	14.45	0.0001	2.79	1.62-4.81	0.36	0.21-0.62
**Correlation**	PFS	7.85	0.005	1.9	1.14-3.16	0.53	0.32-0.88
	OS	11.31	0.0008	2.61	1.53-4.45	0.38	0.23-0.65
**Short Zone Emphasis**	PFS	7.99	0.004	1.87	1.17-3.0	0.53	0.33-0.85
	OS	12.91	0.0003	2.53	1.39-4.62	0.4	0.22-0.72
**Homogeneity**	PFS	3.71	0.05	1.58	1.0-2.49	0.63	0.4-1.0
	OS	5.41	0.01	1.86	1.04-3.33	0.54	0.3-0.96
**TRE**	OS	10.39	0.001	2.34	1.25-4.36	0.43	0.23-0.80

Whole cohort (*n* = 141), 75 demonstrated progressive disease, 54 died. CI = confidence interval, HR = Hazard Ratio, TRE = tissue receptor expression (as a conventional positron emission tomography parameter).

## DISCUSSION

In the present study, we investigated the prognostic value of PET-assessed tumor heterogeneity in patients scheduled for PRRT. Several textural characteristics like Entropy, Skewness, Correlation, Short Zone Emphasis and Homogeneity demonstrated superior diagnostic capability than standard PET parameters such as mean and maximum standardized uptake value (SUV_max_ / SUV_mean_).

Several studies have reported the feasibility of texture analysis for heterogeneity assessment and its prognostic implication for individual patient outcome based on pretherapeutic CT and magnetic resonance imaging, e.g. in non-small cell lung carcinoma or prostate cancer [13–15]. Due to its ability to visualize whole-body tumor burden on a molecular level, PET-based tumor heterogeneity offers certain advantages for assessing intraindividual heterogeneity patterns in tumor biology. Consequently, prognostic capability of imaging-derived intratumoral heterogeneity using ^18^F-FDG but also SSTR-PET/CT has been evaluated in several tumor entities, such as breast, rectal, thyroid or esophageal cancer [4, 6, 16–18]. In NET, given the complexity of various diagnostic procedures and treatment options, early identification of subjects likely to benefit from PRRT would be of great value for individualized treatment tailoring. As demonstrated in the present study, application of textural parameters could be helpful in differentiating high-risk from low-risk groups during PRRT. Strikingly, the 4 parameters Entropy, Correlation, Short Zone Emphasis and Homogeneity provided a significant distinction between responders from non-responders (Table 3, Figures 1 and 2).

In NET, conventional CT-derived therapy response assessment failed to predict disease-related progression or survival [19, 20]. Regarding functional imaging, data is unequivocal: On the one hand, increasing uptake in SSTR scintigraphy (Octreoscan^®^) was reported as a significant predictor of PFS [21, 22]. On the other hand, conventional PET parameters like SUV_max_ failed to predict time-to progression in SSTR-expressing tumor entities [23]. In line with this finding, *Gabriel* and colleagues also demonstrated no benefit from conventional ^68^Ga-DOTATOC PET parameters neither for response nor survival prediction in GEP NET patients scheduled for PRRT [24].

By contrast, heterogeneity parameters demonstrated prognostic value in this study, thereby, outperforming most conventional PET parameters. Of note, only the standard feature TRE proved useful in terms of OS prediction.

Not surprisingly, the cumulative treatment dose correlated significantly with both PFS and OS. This could be regarded as a kind of intra-observer quality control of our study: with increasing progression-free and overall survival, more treatment cycles are performed and, consequently, the administered dose rises.

Interestingly, well-established histology or serum-based parameters of tumor aggressiveness or burden, such as the proliferation index Ki67 (PFS, *p* = 0.09) or CgA (OS, *p* = 0.06), failed to reach prognostic significance. This finding might be influenced by sampling bias: *Couvelard* and colleagues showed that two randomly taken cores from the same metastasis of pancreatic NETs led to a change in grading in half of the cases [25]. Therefore, especially in patients with multiple lesions, non-invasive and reliable whole-body assessment of intra-tumor heterogeneity might yield completely different results [29] and contribute to individualised treatment decisions [30].

Diagnostic imaging of NET is the domain of SSTR agonists as well as ^18^F-FDG PET/CT in case of dedifferentiation [31]. More recently, specific ligands targeting C-X-C motif chemokine receptor 4 (CXCR4) for diagnostic and therapeutic purposes were also introduced [32–37] and in the future, a combination of these above-mentioned radionuclides might also potentially offer additional insight in NET biology and its underlying heterogeneity.

This study has several limitations: As disadvantage of a large multicentric trial, imaging as well as therapeutic protocols might differ from center to center. Especially when dealing with elaborated image analysis as textural features, varieties due to different PET/CT machines and different acquisition protocols may occur. However, using ^18^F-FDG, *Tixier* et al. proved robustness of certain local or regional characterization features (e.g. Entropy), which also reached significance in our analysis [38]. Moreover, we are aware of the multiple testing problem; however, even when we correct for alpha inflation, the majority of tests remain significant.

Though NET is a rare disease with an annual incidence of approximately 5 new cases per 100.000 inhabitants [1], we were able to enroll a cohort of more than 140 patients which underwent PRRT. Therefore, advantages for such a multicentric trial might overcome the disadvantage of different diagnostic or therapeutic protocols. Also the low incidence of this disease complicates prospective reproduction of such analysis, which would be still the next step to achieve more standardized results.

In conclusion, in this multicenter trial enrolling 141 patients, tumor heterogeneity as assessed by baseline SSTR-PET/CT proved prognostic value in PRRT candidates and outperformed common conventional PET parameters. Assessment of intratumoral heterogeneity might significantly contribute to a more individualized patient management and treatment tailoring.

## MATERIALS AND METHODS

All patients gave written and informed consent to the treatment and imaging procedures. The study was approved by local institutional review boards or the requirement for additional approval was waived due to the retrospective character of this study.

### Patient population

In a retrospective cohort, 142 patients (71/141 females (50.4%), 63 ± 11 years, median 64 y, range, 24-83 y) at the four university hospitals of Bonn (*n* = 78), Wuerzburg (*n* = 27), Munich (*n* = 21) and Hannover (*n* = 15) were enrolled. Gastroenteropancreatic (GEP)-NET (including primary tumors of the pancreas, stomach, ileum/jejunum/mesenterium and colon) occurred in 108/141 (76.6%) patients, 15/141 (10.6%) were classified as cancer of unknown primary (CUP), 9/141 (6.4%) had lung NET and the remaining 9 patients (6.4%) suffered from other tumor entities (including meningeoma, hemangioendothelioma, pheochromocytoma, medullary thyroid carcinoma and pituitary tumor). Histological confirmation of the diagnosis was available in every patient. Proliferation index Ki67 ranged between 1-40% with a median of 5%. Chromogranin A (CgA) levels prior to therapy ranged between 35 - 64700 µg/l (median, 571 µg/l). All patients had undergone a number of previous treatments including surgery (*n* = 76/141, 53.9%), sandostatin therapy (*n* = 79/141, 56%), chemotherapy (*n* = 39/141, 27.7%), or external beam radiation therapy (*n* = 15/141, 10.6%).

Clinical and routinely acquired characteristics of the patient cohort including sex, age, prior therapies, site of metastases, CgA, Ki67, and administered activities are given in Table 1.

Radiopeptide therapy was performed according to the Rotterdam protocol as outlined by Kwekkeboom et al. as well as *The joint IAEA, EANM and SNMMI practical guidance* on a compassionate use basis, respectively [11, 19]. A total of 709 treatment cycles (median, 4, range, 1-6) with a median of 7.3 Gigabecquerel (GBq) (range, 0.9-9.0 GBq) per cycle with ^177^Lutetium (^177^Lu)-labeled somatostatin analog (^177^Lu-DOTATATE/- DOTA-D-Phe-Tyr3-octreotide (DOTATOC)) were performed. Imaging including both functional (SSTR-PET/CT) and/or morphologic imaging (CT) modalities was conducted every 3-6 months after PRRT [11]. PFS was defined according to Response Evaluation Criteria in Solid Tumors 1.1 (RECIST 1.1) by serial radiological assessment starting from the time point of baseline imaging and/or according to clinical signs of progression [11, 39. For the calculation of overall survival (OS) the time interval between the pre-therapeutic PET examination and the date of death was used.

### PET/CT imaging

Prior to PRRT, all patients underwent SSTR-PET/CT according to EANM guidelines to assess tumor receptor expression [11]. A median of 124 Megabecquerel (MBq) (range, 61-239 MBq) of ^68^Ga-DOTATATE/-TOC was injected intravenously. After 60 minutes, imaging was performed using the following devices: Bonn, Biograph 2 PET/CT (Siemens Medical Solutions, Erlangen, Germany); Wuerzburg, Biograph 64 (Siemens Medical Solutions, Erlangen, Germany); Munich, Gemini TF PET/CT (Philips Medical, Eindhoven, Netherlands) or Siemens Biograph 64 (Siemens Medical Solutions, Erlangen, Germany); Hannover, Biograph 2 (Siemens Medical Solutions, Erlangen, Germany). All data was reconstructed using iterative algorithms implemented by the manufacturer and depending on the routine protocol of the different medical centers. Scatter and attenuation correction was performed based on the different transmission data.

### Image interpretation

**Figure 3.1:**
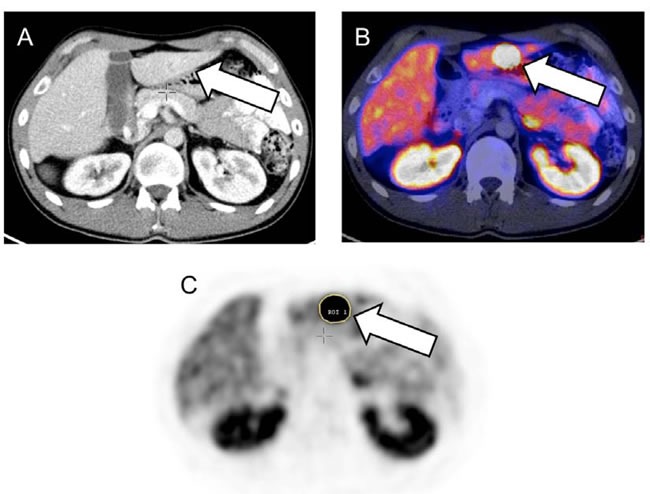
Baseline somatostatin receptor (SSTR) positron emission tomography/computed tomography (PET/CT) of a 24-year old male suffering from metastatic ileum neuroendocrine tumor Liver metastasis can be detected on CT (A) and on SSTR-PET/CT (B) indicated by the arrows. Manual stepwise segmentation of the lesion (arrow) by a region of interest on the PET-only images was performed (C). An overview of investigated heterogeneity parameters can be found in [26, 27], Table 4 and Figure 3.2.

**Figure 3.2:**
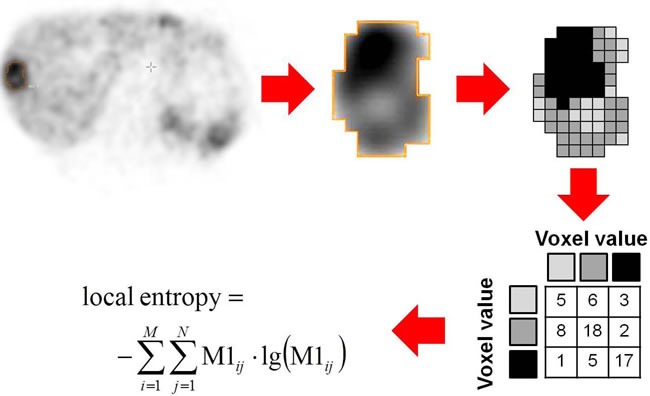
Schematic example of Entropy assessment as one of the heterogeneity parameters Baseline somatostatin receptor positron emission tomography of a subject suffering from liver metastasis of a gastroenteric neuroendocrine tumor. Magnification of a liver metastasis demonstrates intralesional differences in ^68^Ga-DOTA-D-Phe-Tyr3-octreotate (^68^Ga-DOTATATE) distribution. For calculation of Entropy as a second order textural parameter the activity values in the lesion are discretized and a spatial dependence matrix (M1) is created. M1 determines how often a pixel with intensity i finds itself within a certain relationship (e.g. next neighbor in one direction) to another pixel with intensity j in a volume of interest [28].

Analysis of the dataset was performed at the university hospitals of Bonn and Wuerzburg. All image data were transferred to an Interview Fusion Workstation (Mediso Medical Imaging Systems Ltd., Budapest, Hungary). Conventional parameters (such as SUV_mean_/SUV_max_ , TRE)) were derived. TRE is the product of segmented lesion volume times the mean activity uptake, analog to the total lesion glycolysis in ^18^F-fluorodeoxyglucose (^18^F-FDG) PET. For analysis of tumor heterogeneity the largest lymph node, bone and visceral metastases were assessed. For system-based analysis the mean value of these lesions for each individual system was used. For patient-based analysis, the mean value of all segmented lesions per patient was performed. Metastases smaller than 15 mm were not taken into account to avoid partial volume effects. Manual segmentation was executed in combined PET/CT data (Figure 3.1). Several different textural parameters representing intratumoral heterogeneity were derived from every individual lesion and were divided in first order parameters (e.g., Coefficient of variation, COV), second order parameters (e.g., Entropy, Correlation) and higher order parameters (e.g., Grey Level Uniformity, Intensity Variation, Short Zone Emphasis). A detailed description can be found in Table 4, Figure 3.2, [26] and [27]. For comparison to heterogeneity parameters, conventional diagnostic parameters were evaluated: morphologic volume of the lesion, maximum standardized uptake value (SUV_max_), mean SUV (SUV_mean_), and TRE were assessed. The SUV was calculated according to the body weight of the patient. In total, 872 lesions (median, 6, range, 1-23 per patient) were manually segmented.

**Table 4:** Overview of selected textural parameters

Parameter	Order	Description
Coefficient of Variation (COV)	1^st^	A normalized measure of dispersion of a frequency distribution.
Skewness	1^st^	A measure for the extent to which a frequency distribution “leans” to side of the mean value of the distribution.
Entropy	2^st^	Measures grade of derangement, e.g. a homogenous matrix demonstrates low entropy.
Homogeneity	2^st^	A measure for continuous areas of same or similar voxel values in an image or voxel of interest (VOI).
Correlation	2^st^	A measure of intensity linear-dependencies.
Contrast	2^st^	Measures the difference of the grey value when going to the next voxel. It is high when the intensity changes very often between single voxels.
Short Zone Emphasis	3^st^	Measures the distribution of short zones. It is highly dependent on the occurrence of small zones and is expected to be large for fine textures.
Size Variation	3^st^	Describes the variation in the size of different substructures in an image (VOI).

### Statistical analysis

First, Cox multiparametric regression was applied to determine independent prognostic parameters of PFS and OS as well as estimation of HR. Due to multi-colinearity of different textural parameters, only the following parameters were included in the final Cox regression: COV, Skewness, Entropy, Homogeneity, Correlation, Contrast, Short Zone Emphasis and Size Variation. For all independent parameters, ROC analysis was obtained to estimate the optimal cut-off value for the individual parameters to assess progress and OS in the follow-up period. For this purpose, the Youden index was used to maximize the sum of sensitivity and specificity [40]. The area under curve (AUC) was calculated including the exact binominal confidence intervals (95% confidence level). Statistical significance of the prognostic capability was assumed when the critical value of 0.5 was not included in the confidence interval. For the parameters showing such significance, the relationship to both PFS and OS was analyzed using Kaplan-Meier plots. Kaplan-Meier analysis was performed using thresholds established by prior ROC analysis. Differences between Kaplan-Meier curves were evaluated using nonparametric log-rank tests, considering differences with a *p* value smaller than 0.05 to be significant.

Cox analysis was executed using the software package R (version 3.2.4, www.r-project.org). ROC analysis and Kaplan-Meier analysis was performed using MedCalc software (version 12.3.0.0, MedCalc Mariakerke, Belgium).

Parts of this work have been presented at the Annual Meeting of the Society of Nuclear Medicine and Molecular Imaging, 2016, San Diego, United States.

## SUPPLEMENTARY MATERIALS FIGURES AND TABLES


