# Supplementary material for: Survival prediction in patients undergoing radionuclide therapy based on intratumoral somatostatin-receptor heterogeneity
Source: Oncotarget. 2016 Oct 2;8(4):7039–49. doi: 10.18632/oncotarget.12402 (PMC5351689; doi:10.18632/oncotarget.12402)
Supplement: Supplementary file 1 [file oncotarget-08-7039-s001.pdf]

Survival prediction in patients undergoing radionuclide therapy based on intratumoral somatostatin-receptor heterogeneity

Supplementary Material

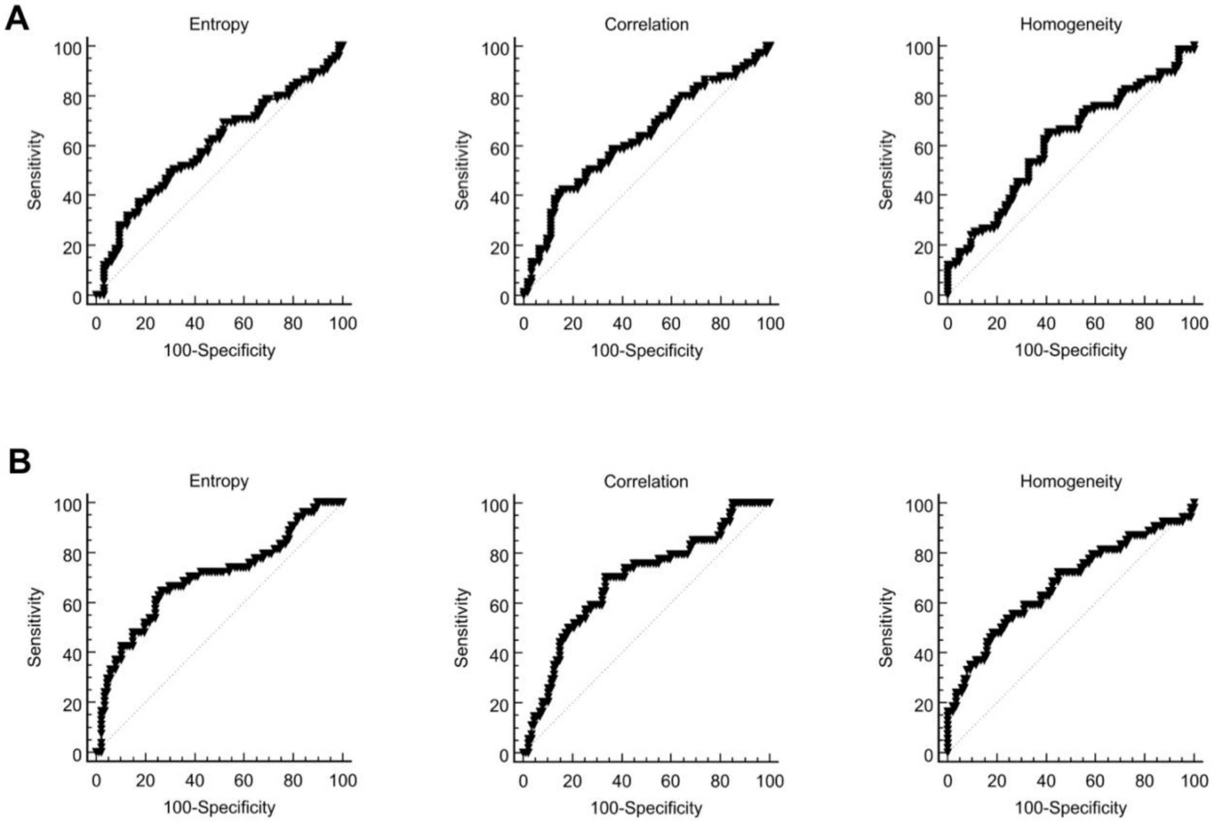

Supplementary Figure a. Receiver Operating Characteristics (ROC) analysis for a) Progression Free (n=139) and b) Overall Survival (n=141) for selected textural parameters being independent according to Cox multiparametric analysis.
